# Serum IL-6 and PTX3 predict severe outcome from COVID-19 in ambulatory subjects: Impact for future therapeutic decisions

**DOI:** 10.1371/journal.pone.0324242

**Published:** 2025-05-27

**Authors:** Josh Poorbaugh, Jonathan T. Sims, Lin Zhang, Ching-Yun Chang, Richard E. Higgs, Ajay Nirula, Robert J. Benschop

**Affiliations:** Eli Lilly and Company, Indianapolis, Indiana, United States of America; Universita degli Studi di Udine Dipartimento di Area Medica, ITALY

## Abstract

SARS-CoV-2 infections lead to a wide-range of outcomes from mild or asymptomatic illness to serious complications and death. While many studies have characterized hospitalized SARS-CoV-2 patient immune responses, we were interested in whether serious complications of SARS-CoV-2 infection could be predicted early in ambulatory subjects. To that end, we used samples from SARS-CoV-2-infected individuals from the placebo arm of the BLAZE-1 clinical trial who progressed to hospitalization or death compared to individuals in the same study who did not require medical intervention and investigated whether baseline serum cytokines and chemokines could predict severe outcome. High-risk demographic factors at baseline, including age, nasal pharyngeal viral load, duration from symptom onset, and BMI provide significant predictive capacity for a hospitalization or death with an AUC of ROC = 0.77. The predictive performance of our outcome modeling increased when baseline serum protein markers were included. In fact, the one-marker model indicated that there were 51 individual proteins (including known markers of inflammation like IL-6, MCP-3, CXCL10, IL-1Ra, and PTX3) that significantly increased the AUC of ROC beyond high-risk patient demographics alone to range between 0.78 to 0.88. Moreover, a two-marker model incorporating levels of both IL-6 and PTX3 further improved the prediction over the addition of a single protein marker to an AUC of ROC = 0.91. While the analytes identified in this study have been well-documented to be altered in SARS-CoV-2 infection, this analysis demonstrates the potential value of their use in predicting hospitalization or death in ambulatory participants infected with SARS-CoV-2 and could guide early treatment decisions.

## Introduction

As of February 2024, there have been over 775 million confirmed cases of COVID-19 globally, including more than 7 million deaths [1]. Infection with the SARS-CoV-2 virus can result in a wide spectrum of symptoms from mild, even asymptomatic, disease to multi-organ failure and death [2]. Strategies for prevention of infection and disease transmission through social distancing, hand washing, etc. were implemented early in the pandemic and are a key component of public health management [3]. However, despite this, and rapid drug development and approval, effective prevention and treatment of the novel coronavirus has been difficult, and new and emerging variants, with enhanced transmissibility and resistance to therapies, have resulted in altered treatment paradigms for some patients [4–6].

In order to combat severe outcomes and treat patients effectively while managing limited hospital system resources, many have sought to identify biomarkers that may predict both therapeutic responses along with disease course and progression. Studies have identified serum IL-10 and testosterone levels in males, serum ACE2 levels, and even an activated neutrophil signature containing a combination of several serum factors (granulocyte colony-stimulating factor (G-CSF) and interleukin-8 (IL-8)) and neutrophil-derived effectors (resistin (RETN), lipocalin-2 (LCN2), and hepatocyte growth factor (HGF)) as predictive of outcome in COVID-19 patients [7–9]. However, these studies all assessed these biomarkers in patients after hospitalization. Predicting poor health outcome before entering the hospital would provide more impact on treatment decisions for the patient and reduce the stress on the hospital system.

To address this, we utilized data and samples from individuals enrolled in the randomized phase 2/3 clinical trial (BLAZE-1, ClinicalTrials.gov Identifier: NCT04427501) that was designed to determine efficacy of a monoclonal antibody therapy in ambulatory subjects who had tested positive for SARS-CoV-2 infection and had one or more mild-to-moderate symptoms as defined according to FDA guidance [10–12]. All participants presented within 3 days of their first positive test result for SARS-CoV-2. Over the course of the trial, some individuals in the placebo arm would experience a COVID-19-Related Hospitalization or Death (CRHD) compared to others who would convalesce without any particular treatment (CC: COVID Convalesce); therefore, we performed a post-hoc analysis on baseline samples from SARS-CoV-2-infected individuals that went on to hospitalization or death compared to matched individuals who did not require medical intervention. From this unique subject cohort, demographic factors, serum cytokines and chemokines were evaluated to determine their ability to predict severe outcome. Importantly, our study identified a pair of early protein biomarkers (IL-6 and PTX-3) able to significantly improve the predictive power for which ambulatory subjects would experience severe outcomes beyond high-risk demographics alone.

## Methods

### Subject sample information

Subjects were recruited into BLAZE-1 as described [10,11]. The original study protocol was reviewed and approved by ethics committees at each of the participating sites. This full list of participating site and ethics committees can be found for BLAZE-1 using ClinicalTrials.gov Identifier: NCT04427501. Briefly, participant demographics were collected at study entry with each subject categorized with mild or moderate COVID-19 as defined per US Food and Drug Administration guidance and included symptoms such as fever, cough, sore throat, malaise, headache, muscle pain, gastrointestinal symptoms, and shortness of breath with exertion. Furthermore, serum was collected at study entry from ambulatory individuals within 3 days of their first positive test result for SARS-CoV-2. Individuals were selected from the placebo arm of the trial and divided into the CRHD group (n = 56) (with a COVID-19-related hospital visit (n = 43) or died (n = 13)) and compared to the CC group (n = 132), the participants who recovered from the SARS-CoV-2 infection without treatment. Sample selections were matched for baseline nasopharyngeal viral load levels and days since symptom onset using data accessed after October 14, 2020 following review of informed consent documentation. The authors had no access to information that could potentially identify individual participants at any point during or after the collection of data ensuring the confidentiality of the study subjects. Serum from 49 age/sex-matched healthy, uninfected controls were also collected for comparison.

### Viral load determination

Viral load was measured by nasopharyngeal swab followed by quantitative RT-PCR reaction [10,13]. Viral load data are based on the cycle threshold and calculated as an arbitrary unit. The primer sequences for the RT-PCR assay have been reported previously [14].

### Serum protein determination

Serum was analyzed with the Olink Inflammation I (95302) and Cardiovascular II (95500) proximity extension array technology panels (Uppsala, Sweden), each measuring 92 analytes, according to manufacturer protocols. The levels of analyte-specific, DNA amplicons for all 184 analytes were quantified for each sample on a Fluidigm BioMark HD (San Francisco, CA). The unit of measure, calculated from cycle threshold (Ct) values and expressed on a log2 scale, is represented as normalized protein expression (NPX) values.

### Statistical analysis

The Olink NPX results were used to create volcano plots comparing the differences between subject groupings (all patients vs Healthy control, CRHD vs CC and hospital visit vs death). Log2 fold-changes were calculated using the comparisons identified while pairwise comparisons across all markers were used to calculate adjusted p-values. For individual plots of selected markers from healthy and COVID-19 subjects, one-way ANOVA with post-hoc Tukey analysis was performed for multiplicity comparison testing (GraphPad Prism, version 9.5.0(730)).

Logistic regression was used to predict CRHD vs CC outcome with leave-one-out cross validation and all predictive model performance results were obtained from this leave-one-out procedure. The model with demographic variables (i.e., baseline normalized viral load, duration of symptom onset, BMI, and age) was considered the baseline model (S1 Table). The improvement in performance of the baseline model by adding biomarker(s) to demographic variables was evaluated using area under the curve (AUC) of the receiver operating characteristic curve (ROC). A higher AUC value indicates better model performance (from 0 to 1). To be specific, for each biomarker, the leave-one-out ROC analysis were repeated 100 times with random shuffles of the biomarker data that preserving correlation structure (i.e., permutation test), and permutated p-value equals probability of observed AUC < 100 permutated AUCs. There is significant improvement of predictive performance if (1) permutated p-value from the permutation test is less than 0.05, and (2) AUC is greater than 0.77 and increased from 0.77 by at least 0.02. Analyses were conducted in R and results represented using ROC curves [15].

To evaluate the benefit of adding additional baseline protein biomarker to boost the predictive performance, a “forward-selection” method was used. For a (p + 1)-biomarker predictive model, permutation test (as described in previous paragraph) was conducted 100 times. There is significant improvement of predictive performance if (1) permutated p-value from the permutation test is less than 0.05, and (2) AUC of a (p + 1)-biomarker predictive model is greater than the AUC of the p-biomarker predictive model with a decent increase by at least 0.02.

## Results

In the summer of 2020, clinical evaluation of the SARS-CoV-2 monoclonal antibody treatment, Bamlanivimab, was conducted in an ambulatory population as part of the BLAZE-1 study. While Bamlanivimab significantly altered disease progression in treated individuals, individuals treated with placebo experienced a low rate of COVID-19-related hospital visits or death (CRHD) [10]. Following completion of the study, these individuals with CRHD outcomes (n = 56) were compared with placebo-treated individuals who fully convalesced from COVID-19 (CC; n = 132) to retrospectively determine whether baseline immune parameters could predict outcome above and beyond well-known demographic characteristics.

As seen in Table 1, subjects were classified as either mild or moderate within the CRHD and CC subgroups. In the CC group there were 132 mild individuals along with 25 moderate subjects. 32 persons from the CRHD group were categorized as mild while 24 were moderate. Importantly, there were 13 total deaths and 43 hospitalizations within the CRHD subgroup. Of these, 9 with a mild baseline severity score died, while 23 were hospitalized. Four with a moderate baseline severity score also died and 20 were hospitalized. Therefore, the mild or moderate classification system does not lead to stratifying individuals by potential future outcome by itself. While studies have characterized differentially expressed serum cytokines in hospitalized patients, immune dysregulation in ambulatory subjects has been less studied [16–18]. Our unique cohort allows for a chance to evaluate if serum cytokines and chemokines could predict severe outcome in ambulatory subjects.

**Table 1:** Characteristics of study populations.

Subject Characteristics	CRHD (n = 56)		CC (n = 132)	
	Mild	Mod	Mild	Mod
n	32	24	107	25
Age (Mean Years; Range)	67 (34-92)	54 (36-77)	53 (19-81)	52 (26-72)
BMI (Mean; SD)	33.5 (+/-9.7)	38.7 (+/-7.5)	33.2 (+/-6.9)	34.4 (+/-8.0)
Baseline Viral Load (Mean; SD)	7.8 (+/-1.3)	7.4 (+/-1.4)	7.0 (+/-2.2)	7.0 (+/-1.8)
Days since symptom onset (Mean; SD)	4 (+/-2)	3 (+/-2)	5 (+/-3)	5 (+/-3)

### High-risk subject demographics

**Fig 1:**
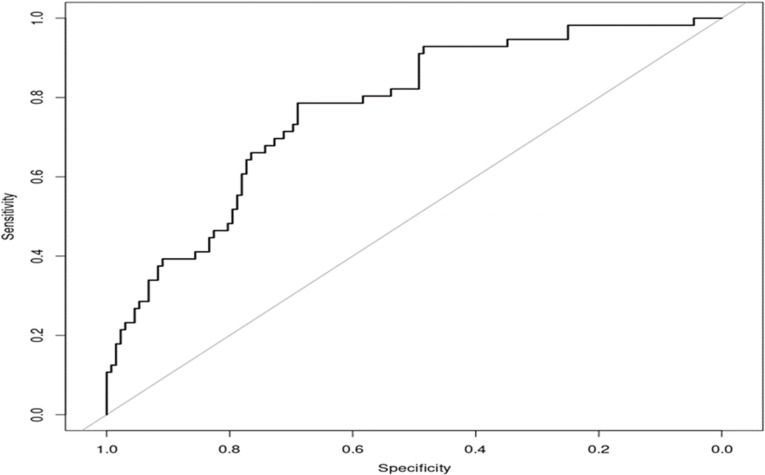
Predictive ability of high-risk demographics alone is moderately related to outcome in ambulatory subjects. High-risk demographics (age, BMI, baseline viral load and days from symptom onset) predict severe outcome from SARS-CoV-2 infection with an AUC of ROC = 0.77.

In clinical practice, general consideration of high-risk demographics including BMI, presence of a preexisting condition, and age have already been useful for subject classification [19,20]. As observed in Table 1, baseline demographic characteristics of the CRHD and the CC groups were comparable between the groups. High-risk demographic parameters (including viral load at baseline) alone were able to predict which individuals progressed to more severe outcomes from an ambulatory setting (AUC of ROC = 0.77) (Fig 1). Given that subjects who died were split across the moderate and mild subgroups, we wanted to investigate if additional factors could have a significant improvement in the ROC curve analysis.

### Assessment of serum biomarkers

**Fig 2:**
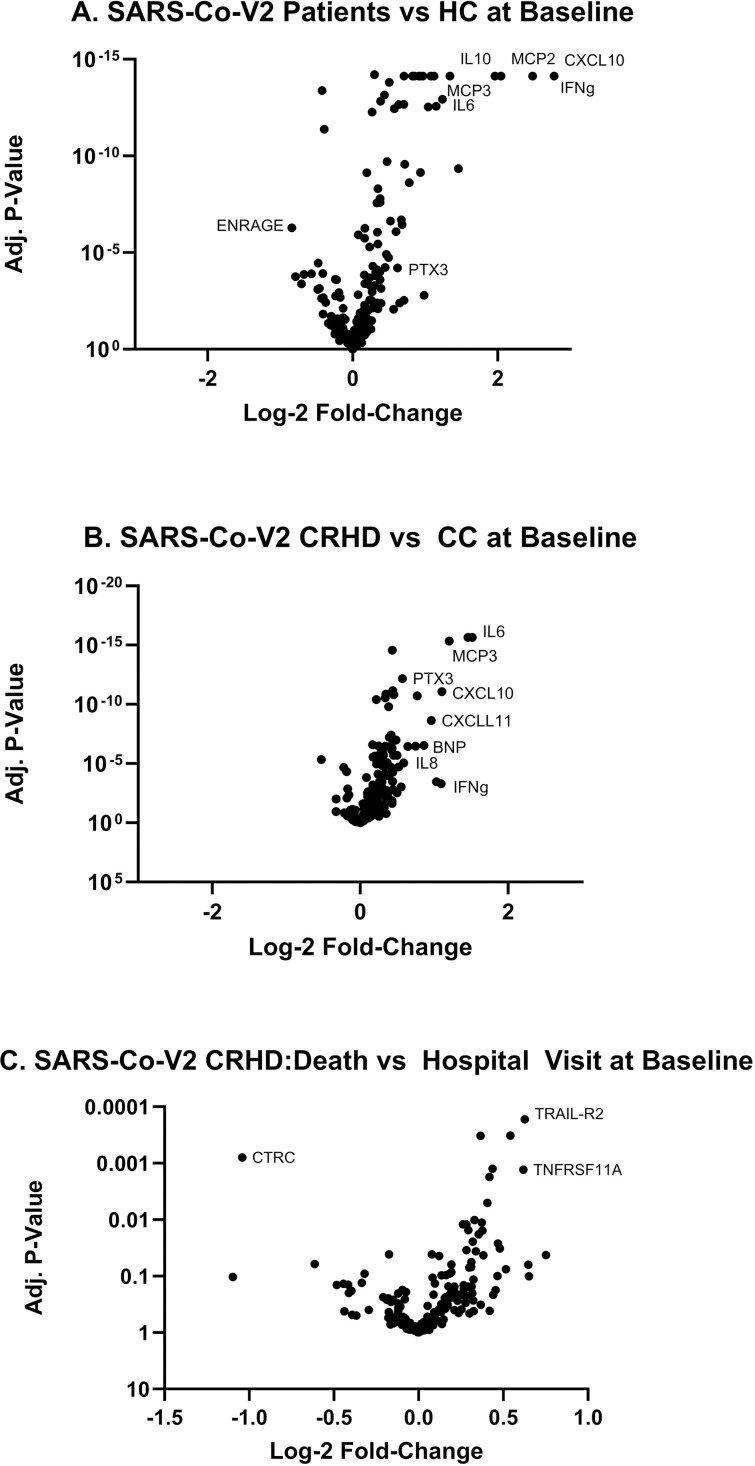
Analysis of baseline serum proteins in SARS-CoV-2 infected subjects who went on to those requiring a hospital visit or died compared to healthy volunteers. Volcano plots demonstrate the overall landscape of protein expression changes in baseline serum samples of (A) SARS-CoV-2-infected individuals (N = 186) vs healthy volunteers (N = 50), (B) CRHD (N = 56) vs CC (N = 132), and (C) those that would die (N = 13) compared those that had a hospital visit (N = 43).

Before comparing serum protein levels between the CRHD and CC cohorts, we first assessed any differences between all SARS-CoV-2-infected individuals and age/sex-matched healthy controls (HC). The volcano plot in Fig 2A, demonstrates an expression pattern consistent with previous studies of infected individuals [16,18]. IFNγ, CXCL10, and IL-6 are among many elevated proteins at baseline in all COVID-19 positive subjects relative to healthy subjects. However, when comparing the CRHD and CC SARS-CoV-2 infected groups several analytes were found to be significantly higher in the CRHD group. Levels of BNP and IL-8 were significantly elevated in the CRHD group compared to the CC group, while IL-6, MCP-3, and CXCL10 continued to be further elevated in the CRHD group indicating a more reactive immune response at baseline (Fig 2B). We further separated individuals in the CRHD group that proceeded to death from those that required a hospital visit (Fig 2C). In this comparison, baseline differences had smaller fold-differences and larger p-values, most likely due to our reduced subject numbers for these subgroups. Still, markers like TRAIL-R2, TNFRSF11A, and CTRC were significantly modulated in those that died as a result of SARS-CoV-2 infection.

**Fig 3:**
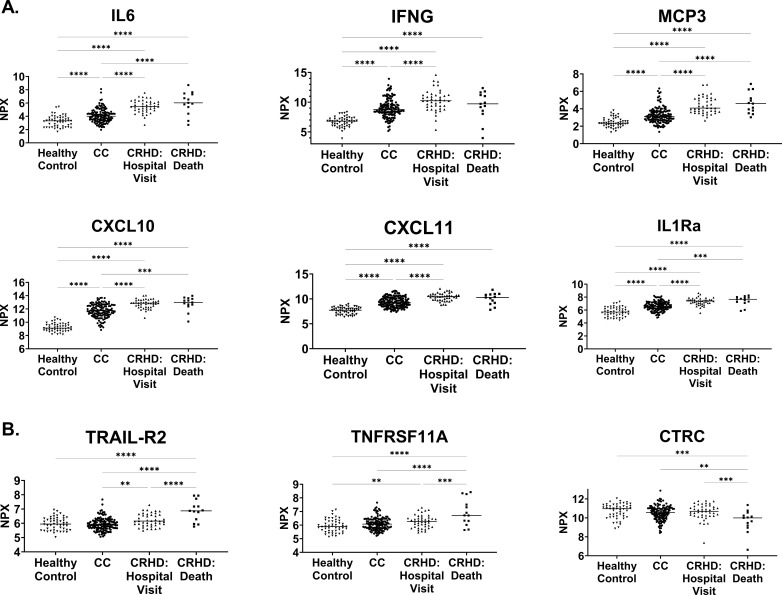
Inflammatory cytokine profiles grouped by severity. Protein assays were performed to assess serum cytokine and chemokine levels across healthy controls (N = 50), CC (N = 132), and the individual CRHD: Hospital Visit (N = 43) and CRHD: Death (N = 13) subgroups. In panel A, these proteins are elevated in CRHD vs CC individuals while in panel B, significant differences were observed within the CRHD subgroup, between those requiring a hospital visit and those that died.

Levels of significantly altered inflammatory cytokines (fold changes > 1.5 and an adjusted p-value < 1E-3) from the baseline CRHD vs CC group comparison were plotted by subject severity (Fig 3A). As expected, these highly dysregulated proteins were significantly elevated relative to the HC population, demonstrating the previously published pattern of an increased proinflammatory profile as a result of SARS-CoV-2 infection [21]. However, these important serum proteins demonstrated significantly higher levels in the baseline samples in individuals that would require a hospital visit or proceed to death compared to those who would recover normally. Of these, IL-6, IFNγ, and MCP-3 were the most significantly elevated in the CRHD group compared to those who would convalesce (fold changes > 2 with an adjusted p-value < 1E-3). Interestingly, none of these markers significantly distinguished between the outcomes of hospitalization versus death with IFNγ, in fact, not even significantly different between CC and the CRHD death subgroup. Other proteins, such as TRAIL-R2, TNFRSF11A, and CTRC, were differentially expressed at baseline in the CRHD cohort relative to the CC cohort (adjusted p-value < 1E-3 for all analytes) (Fig 3B) and, yet, indistinguishable in SARS-CoV-2 positive subjects that convalesce compared to healthy controls [18]. Most interestingly, these proteins were significantly altered in the CRHD death subgroup, highlighting that some baseline protein levels hold potential to possibly predict severe outcomes.

### Addition of serum protein biomarkers to ROC analysis

**Fig 4:**
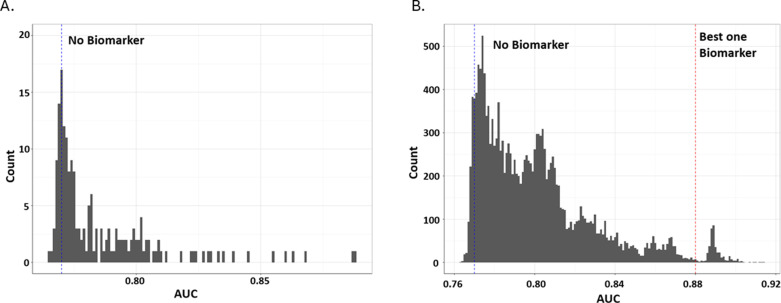
Iterative analysis of Serum Biomarkers to Improve ROC Prediction. Iterative addition of up to two serum biomarker levels from the protein panels resulted in significant improvements to the AUC of ROC. 51 individual markers improved the AUC of ROC compared to baseline high-risk demographics alone (A), with fewer combinations of 2 markers showing improvement over single molecule additions (B).

To improve the predictive model beyond baseline high-risk demographics alone (Fig 1), baseline serum protein biomarkers were incorporated for modeling. Fifty-one individual proteins at baseline demonstrated a significantly increased AUC of the ROC curve when added to the baseline high-risk demographics (S2-S4 Table and Fig 4A). Although previous proteomic analysis of SARS-CoV2-infected individuals had revealed elevated expression levels of serum IL-6, CXCL10, IFNγ, MCP-3, and CXCL11 relative to healthy controls, these studies did not evaluate the predictive ability to the severity of outcome of these proteins [17,18,21].

**Fig 5:**
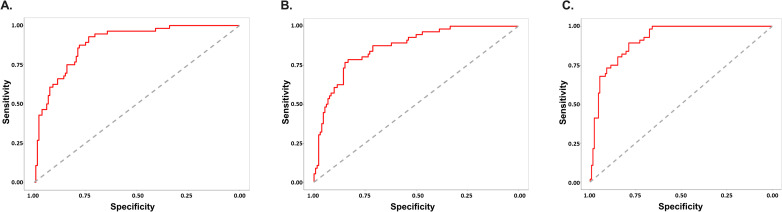
Combination of IL-6 and PTX3, in addition to subject high-risk demographics, improve the AUC of the ROC curve and increase predictive power of the model to identify subjects who will have severe SARS-CoV-2 outcomes. Subject high-risk demographics, combined with baseline IL-6 (A) and PTX3 (B) serum levels alone or in combination (C) increase the ability to predict severe outcome from SARS-CoV-2 infection. IL-6 AUC of the ROC curve is 0.89 (95% confidence interval (CI) 0.84-0.94) and PTX3 AUC of ROC curve is 0.86 (95% CI 0.81-0.92). IL-6 and PTX3 combination AUC of ROC is 0.91 (95% CI 0.87-0.95).

The large number of proteins with added predictive performance prompted further evaluation to determine if two or more proteins could add to the predictive power (Fig 4B). IL-6 was the most predictive single analytes improving the AUC of the ROC to 0.89 (Fig 5A). A “forward-selection” method was used to determine which additional baseline protein biomarkers need to be included in addition to IL-6. The combination of baseline IL-6 and PTX3 generated the highest AUC of the ROC curve (AUC = 0.91) when added to subject high-risk demographics (Fig 5C). This was unexpected as the single addition of PTX-3 alone to high-risk subject demographics only increased the AUC of the ROC to 0.85 (Fig 5B). Intriguingly, IL-6 was more prominently dysregulated relative to PTX-3 in SARS-CoV-2 subjects (Fig 2A) and the CRHD subject group (Fig 2B). Though the upregulation of PTX-3 was of lower magnitude than other commonly reported inflammatory analytes (Fig 3A), these changes were highly impactful in ROC prediction of subject outcome (Fig 5). The two proteins together provide the best predictive model of severe outcome to SARS-CoV-2 infection than either alone when added to the subject high-risk demographics in an ambulatory population. The combination of three or more proteins no longer improved the AUC of the ROC curve analysis over the combination of IL-6 and PTX3.

## Discussion

Subject outcomes and responses to SARS-CoV-2 infection are heterogeneous and range from mild to severe symptoms that resolve without medical intervention, to hospital visits and death [22]. Assessment of recently infected individuals in an ambulatory setting currently lacks standardization which can make treatment decisions more difficult without objective biomarkers indicating those who will have a severe outcome [23]. In fact, subject classification at disease presentation can vary due to distinct severity assessments utilized globally. For example, the US National Institutes of Health (NIH) and the World Health Organization (WHO) have proposed different sets of criteria to categorize subjects by severity based on the saturation of oxygen (SpO2) threshold [24–26]. The purpose of this study was to characterize subject responses in SARS-CoV-2 infection and identify biomarkers that help predict outcome in an ambulatory population.

The BLAZE-1 study entry criteria included one or more mild symptoms with presentation within three days of a positive direct antigen or PCR test for SARS-CoV-2 infection [10]. Serum protein analysis at this early stage of infection provided a unique opportunity for identification of a predictive biomarker. The CRHD and CC subjects had significantly higher levels in a variety of proinflammatory markers compared to age/sex-matched healthy controls including IL-6, MCP-3, CXCL9, CXCL10 and others. Interestingly, we found early levels of TRAIL-R2, TNFRSF11A, and CTRC to be statistically elevated in subjects who would die from COVID-19 complications. These proteins may indicate dysregulation of apoptotic cell death or an anti-inflammatory pathway even at this relatively early stage of SARS-CoV-2 infection. TRAIL-R2 has been previously identified as being elevated as early as 12-hours post-SARS-CoV-2 infection, can drive apoptotic pathways and holds predictive power of severe outcome including death in hospitalized subjects [27,28]. Additionally, studies from our group found that the levels of TRAIL-R2 inversely correlated with blood oxygen saturation (SpO2) and positively correlated with disease severity (WHO Ordinal Scale) at hospital admission for COVID-19 subjects [18]. TNFRSF11A has been shown to induce apoptosis in at least some cell types [29]. CTRC is an exocrine pancreas protease previously found to be reduced at day 7 in subjects who progress toward death from SARS-CoV-2 infection [21,30]. Although these factors did not increase the AUC of the ROC curve to predict outcome, it could be because they were only significantly elevated in the subjects who died, and the numbers were too small in our study to account for a pronounced effect.

Our study identified that 51 separate serum proteins increased the predictive capacity of a severe outcome over baseline high-risk demographics alone. While several proteins were differentially regulated to a greater degree than PTX (Fig 2B), of these factors, serum IL-6 and PTX3 stood out, as their combination resulted in the best AUC of the ROC curve (0.91, range 0.87–0.95, p < 0.001). Individually, serum IL-6 was identified early in the COVID-19 pandemic and has been reported to be increased in severe vs non-severe COVID-19, an independent predictor of ICU admission, and higher in COVID-19 deaths than survivors [16,31]. PTX3, likewise, has been identified as a predictor of 28-day mortality in hospitalized subjects with COVID-19 [32]. However, the combination of serum IL-6 and PTX3 together provides a better predictive model of severe outcome to SARS-CoV-2 infection than either alone when added to the subject high-risk demographics in this ambulatory population.

The results presented in this study identify biomarker differences between ambulatory participants experiencing a COVID-19 related hospital visit or death and those that convalesce on their own. While an IL-6 clinical assay is available for rapid quantitation in a subject setting [33], there is not currently a test for PTX3. While PTX3 is related to CRP, it may provide better utility as a biomarker for COVID-19 infection [34]. Our study is limited as it relies on a relatively low number of subjects that died and has not been validated in an independent cohort. As clinical trials are run, it would be important to assess these markers in a prospective cohort. Additionally, our cohort and biomarker data are specific to the dominant variants at that time and our samples were collected in the summer of 2020, when D614G was prevalent [35]. Since previous reports comparing the inflammatory signatures induce by Wuhan, Delta and Omicron variants are overall comparable, it is reasonable to assume that the IL-6/PTX3 relationship would continue as new variants emerge, but this warrants further validation in future cohorts [36]. Lastly, while contemporaneous studies in hospitalized subjects have reported that higher nasal pharyngeal viral load can predict mortality, we did not investigate the predictive power of this parameter because we balanced our two study populations for similar viral loads [37]. Moreover, ambulatory subjects who died did not have a significantly higher viral load within three days of symptom onset compared to those who would convalesce. Instead, our study identified altered serum protein expression patterns at baseline in participants who would go on to develop severe outcomes even when viral loads were comparable. It would be interesting in future studies to evaluate our protein biomarker combination in individuals with different viral loads.

In conclusion, the development of a test for IL-6 and PTX3 to predict CRHD in SARS-CoV-2-positive ambulatory individuals could dramatically improve and/or standardize treatment decisions for those affected. Our unique cohort of ambulatory subjects provides, for the first time, a sample set comparable to data from hospitalized subjects, showing alterations of similar proteins like TRAIL-R2, IL-6, MCP-3 and others at baseline before individuals are experiencing severe symptoms [21]. Add to this the predictive capacity of the IL-6/PTX-3 biomarker combination and there is potential to establish cut points for a novel clinical assay for these analytes to improve the standard of care and treatment decisions.

## Supporting information

S1 Table Logistic regression results. Table containing the odds ratios (OR) and confidence intervals (CI) for the variables included in the initial logistic regression model.
(PDF)

S2 Table Proteins enhancing AUC for severe COVID-19 prediction. List of proteins that improved the AUC of the ROC curve when added to baseline high-risk demographics to predict severe COVID-19 outcome.
(PDF)

S3 Table Individual subject level data. (XLSX)

S4 Table Individual protein level data reported in Figs 2 and 3. (XLSX)
