# Supplementary material for: Serum IL-6 and PTX3 predict severe outcome from COVID-19 in ambulatory subjects: Impact for future therapeutic decisions
Source: PLoS One. 2025 May 27;20(5):e0324242. doi: 10.1371/journal.pone.0324242 (PMC12111355; doi:10.1371/journal.pone.0324242)
Supplement: S1 Table — Table containing the odds ratios (OR) and confidence intervals (CI) for the variables included in the initial logistic regression model. (PDF) [file pone.0324242.s001.pdf]

		95% CI	
	**OR**	**LB**	**UB**
**Baseline normalized viral load**	1	0.8	1.3
**Duration of symptom onset**	0.7	0.5	0.9
**BMI**	1.1	1	1.2
**Age**	1.1	1	1.1
**PTX3**	5.7	2.4	14.7
**IL6**	2.5	1.7	3.9
