# Supplementary material for: Serum IL-6 and PTX3 predict severe outcome from COVID-19 in ambulatory subjects: Impact for future therapeutic decisions
Source: PLoS One. 2025 May 27;20(5):e0324242. doi: 10.1371/journal.pone.0324242 (PMC12111355; doi:10.1371/journal.pone.0324242)
Supplement: S2 Table — List of proteins that improved the AUC of the ROC curve when added to baseline high-risk demographics to predict severe COVID-19 outcome. (PDF) [file pone.0324242.s002.pdf]

S2 Table. List of proteins that improved the AUC of the ROC curve when added to baseline high-risk demographics to predict severe COVID-19 outcome.

Biomarker	AUC of ROC	AUC Lower Bound	AUC Upper Bound	Adjusted p-value
**IL6**	0.89	0.84	0.94	<0.0001
**MCP-3**	0.87	0.82	0.92	<0.0001
**PTX3**	0.86	0.81	0.92	<0.0001
**CXCL10**	0.86	0.80	0.91	<0.0001
**IL-1Ra**	0.86	0.80	0.91	<0.0001
**PGF**	0.84	0.79	0.90	<0.0001
**CXCL11**	0.84	0.78	0.90	<0.0001
**TNFRSF10A**	0.84	0.78	0.89	<0.0001
**PD-L2**	0.83	0.77	0.89	<0.0001
**LIF-R**	0.83	0.77	0.89	<0.0001
**IL-4RA**	0.83	0.77	0.89	<0.0001
**PRELP**	0.83	0.76	0.89	<0.0001
**PARP-1**	0.82	0.76	0.89	0.0001
**IFNγ**	0.82	0.76	0.89	0.0002
**DCN**	0.82	0.76	0.89	0.0001
**OPG**	0.82	0.75	0.88	0.0005
**IL10**	0.81	0.75	0.88	0.0002
**SLAMF7**	0.81	0.75	0.87	0.0018
**AMBP**	0.81	0.75	0.87	0.0004
**TRAIL-R2**	0.81	0.75	0.87	0.0001
**VEGFA**	0.81	0.74	0.87	0.0003
**FGF-23**	0.81	0.74	0.87	0.0001
**HSP 27**	0.81	0.74	0.87	0.0090
**CTSL1**	0.81	0.74	0.87	0.0005
**CXCL1**	0.80	0.74	0.87	0.0013

**ADM**	0.80	0.73	0.87	0.0058
**PD-L1**	0.80	0.74	0.87	0.0006
**CASP-8**	0.80	0.74	0.87	0.0088
**LIF**	0.80	0.74	0.87	0.0141
**Gal-9**	0.80	0.74	0.87	0.0039
**CSF-1**	0.80	0.74	0.87	0.0013
**CDCP1**	0.80	0.73	0.87	0.0061
**IL16**	0.80	0.73	0.87	0.0113
**MMP7**	0.80	0.74	0.86	0.0004
**TWEAK**	0.80	0.73	0.86	0.0051
**TRANCE**	0.80	0.73	0.86	0.0025
**CD4**	0.80	0.73	0.86	0.0022
**hOSCAR**	0.80	0.73	0.86	0.0078
**BNP**	0.80	0.73	0.86	0.0042
**CX3CL1**	0.80	0.73	0.86	0.0014
**MERTK**	0.79	0.73	0.86	0.0015
**MCP-1**	0.79	0.72	0.86	0.0134
**CD40**	0.79	0.73	0.86	0.0007
**KIM1**	0.79	0.72	0.86	0.0021
**IL7**	0.79	0.72	0.86	0.0049
**IL-27**	0.79	0.72	0.86	0.0118
**PAR-1**	0.79	0.72	0.86	0.0035
**GH**	0.79	0.72	0.86	0.0062
**SLAMF1**	0.79	0.72	0.86	0.0127
**TM**	0.78	0.72	0.85	0.0049
**AGRP**	0.78	0.71	0.85	0.0043
